# Supplementary material for: Delivery of a post-natal neonatal jaundice education intervention improves knowledge among mothers at Jinja Regional Referral Hospital in Uganda
Source: PLoS One. 2024 Apr 4;19(4):e0301512. doi: 10.1371/journal.pone.0301512 (PMC10994313; doi:10.1371/journal.pone.0301512)
Supplement: S1 File — (DOCX) [file pone.0301512.s001.docx]

**NNJ Knowledge Questions (Total points possible: 25)**

1. What are some danger signs of the newborn baby that you know? (1 point for each answer, up to 5 points – open ended question, do not give mothers options)
2. Difficulty breathing (chest wall in-drawing, fast breathing)
3. Poor feeding (refusing to eat, not eating enough)
4. Yellow discoloration of the skin (especially palms of hands and soles of feet)
5. Convulsions (shaking)
6. High or low body temperature (hot skin, cold skin)
7. Other………………………………
8. Jaundice causes which of the following? (1 point):
9. Difficulty breathing.
10. Yellowing of the skin and eyes.
11. Swelling of the baby’s face.
12. Baby passing watery stool.
13. Is jaundice a common problem in babies? (1 point)
    1. Yes
    2. No
    3. Don’t know
14. A baby can develop jaundice as early as 24 hours of life (1 point).
15. Yes
16. No
17. Don’t know
18. Can neonatal jaundice be dangerous to the baby? (1 point)
19. Yes
20. No
21. Don’t know
22. Which of the following are signs of neonatal jaundice? (More than one may apply, 1 point each, up to 4 points)
23. Smiling.
24. Yellow skin and/or eyes.
25. Poor feeding.
26. Convulsions.
27. Excessive crying
28. Where would you check on the baby for neonatal jaundice? (More than one may apply, 1 point each, up to 3 points)
29. Hair
30. Eyes
31. Sweat
32. Skin
33. Mouth
34. Select the things make a baby at high risk for developing jaundice? (More than one may apply, 1 point each, up to 4 points)
35. Small or premature baby.
36. Excessive crying.
37. Sibling who had jaundice as a baby
38. Infection.
39. Brown skin
40. Not eating enough
41. Can foods or medications taken by the mother contribute to jaundice in the baby? (1 point)
    1. Yes
    2. No
    3. Don’t Know
42. If a baby develops jaundice within the first day of life, or has jaundice that lasts longer than 7 days, what should you do? (1 point)
    1. Nothing
    2. Expose baby to sunlight
    3. Bring baby to the hospital
    4. Feed the baby water
43. What are some of ways that the hospital will treat jaundice? (Open ended, do not give mothers the answers, 1 point each, up to 2 points)
    1. Bright lights (phototherapy)
    2. Increase baby’s intake (IV fluids, continued feeding)
    3. Exchange blood transfusion
    4. Medications
44. If your baby is admitted to the hospital for jaundice, what is the most important thing for you to do while your baby is in the hospital? (1 point)
    1. Give the baby water
    2. Do nothing
    3. Continue breastfeeding

**Knowledge points scoring guide** (Total score points possible = 25).

| **Question number** | **Maximum score points** | **Correct responses** |
| --- | --- | --- |
| 1 | 5 | All, (Open ended question, do not give mothers options) |
| 2 | 1 | b |
| 3 | 1 | a |
| 4 | 1 | a |
| 5 | 1 | a |
| 6 | 4 | b, c, d & e |
| 7 | 3 | b, d & e |
| 8 | 4 | a, c, d & f |
| 9 | 1 | a |
| 10 | 1 | c |
| 11 | 2 | a, b, c, d (open ended, do not give mothers the answers) |
| 12 | 1 | c |
